# Notices of New Advertisements

**Published:** 1878-06-20

**Authors:** 


BEDFORD ALUM AND IRON SPRINGS.

Notice the advertisement of the above celebrated waters. These springs possess real merit. The water and mass may be obtained in Atlanta, of Messrs. Samuels, Pemberton & Reynolds, R. Holt, and Pinson & Peacock, druggists.

WM. R. WARNER & CO.—New and interesting advertisement, by the house of Wm. R. Warner & Co., appears in the present number of our journal.

McKESSON & ROBBINS' new advertisement, attesting the purity and reliability of their Quinine preparations, should be carefully read.

